# Relay model for recruiting alcohol dependent patients in general hospitals - a single-blind pragmatic randomized trial

**DOI:** 10.1186/s12913-016-1376-8

**Published:** 2016-04-14

**Authors:** Anne-Sophie Schwarz, Randi Bilberg, Lene Bjerregaard, Bent Nielsen, Jes Søgaard, Anette Søgaard Nielsen

**Affiliations:** RESCueH studies, Unit of Clinical Alcohol Research, Clinical Institute, Faculty of Health Sciences, University of Southern Denmark, Odense, Denmark; The Danish Cancer Society and Aarhus University, Faculty of Health Science, Institute of Clinical Medicine, Department of Clinical Epidemiology, Copenhagen, Denmark

**Keywords:** Alcohol Use Disorder, Recruitment, Health promotion, Health economics, Register study

## Abstract

**Background:**

A large proportion of the Danish population consumes more than the officially recommended weekly amount of alcohol. Untreated alcohol use disorders lead to frequent contacts with the health care system and can be associated with considerable human and societal costs. However, only a small share of those with alcohol use disorders receives treatment. A referral model to ensure treatment for alcohol dependent patients after discharge is needed. This study evaluates the i) cost-effectiveness ii) efficacy and iii) overall impact on societal costs of the proposed referral model - The Relay Model.

**Method/Design:**

The study is a single-blind pragmatic randomized controlled trial including patients admitted to the hospital. The study group (*n* = 500) will receive an intervention, and the control group (*n* = 500) will be referred to treatment by usual procedures. All patients complete a lifestyle questionnaire with the Alcohol Use Disorders Identification Test embedded as a case identification strategy. The primary outcome of the study will be health care expenditures 12 months after discharge. The secondary outcome will be the percentage of the target group, who 30 days after discharge, reports at the alcohol treatment clinics. In order to analyse both outcomes, difference-in-difference models will be used.

**Discussion:**

We expect to establish evidence as to whether The Relay Model is either cost-neutral or cost-effective, compared to referral by usual procedures.

**Trial registration:**

https://register.clinicaltrials.gov/by identifier:

RESCueH_Relay NCT02188043 Project Relay Model for Recruiting Alcohol Dependent Patients in General Hospitals (TRN Registration: 07/09/2014)

## Background

Compared to other countries the Danish population has a large intake of alcohol per capita, and almost all adolescents and adults in Denmark drink alcohol [1]. It is estimated that approximately 20 % of the adult population are heavy drinkers consuming >14/21 drinks/week (women/men), 14 % have a harmful alcohol use, and 3 % are dependent drinkers [2]. In Denmark the vast majority of services offered to patients suffering from alcohol use disorders are publicly funded and provided by a highly diverse group of about 60 specialized outpatient clinics, treating nearly 16,000 patients per year [3]. This is a small fraction of the estimated 140,000 alcohol dependent Danes [2], who needs specialized alcohol treatment.

Alcohol use disorders are associated with considerable societal costs and welfare losses to the Danish population. Annual societal costs include excess health care consumption, income loss compensations, children and youth intervention costs [3], production loss due to alcohol related short and long-term inability to work and premature death [4], and costs of alcohol related criminal activity and traffic accidents [5].

Carefully designed prevention and treatment interventions for alcohol use disorders can, if put into scale, help thousands of Danes and in addition generate economic benefits in terms of savings in the costs to society, listed above. Even if specialized treatment delivery itself is costly, it may generate sufficient savings to become cost-effective and perhaps even net-cost-saving [6]. A first approach may be to identify and recruit patients from general hospitals.

Alcohol use disorders are responsible for considerable physical morbidity and accidents, and many patients with alcohol problems are admitted to the general hospitals [7, 8]. In a systematic review of hospital screening studies the proportion of inpatients with alcohol use disorders was found to range between 16 and 26 % [9]. The prevalence is influenced by many factors: e.g. patient population, definitions, and assessment methods. The prevalence of dependence, determined by a diagnostic instrument, among patients in general hospitals is very high [10].

The incidence of patients with alcohol-related diseases is greatest in the departments receiving patients with the following conditions: falls, collapse, head injuries, assaults, gastrointestinal problems, unwell, neurological-psychiatric problems, cardiac symptoms and accidents [11, 12]. In Denmark such patients will typically be admitted to gastrointestinal (liver disease, oesophagitis, gastritis, pancreatitis), neurological (including seizures, head injure, stroke, peripheral neuropathy and vascular disease) and orthopaedic departments (falls, assault, accidents).

Many studies have examined brief interventions for patients in general hospitals [13–15], but it is not well established if alcohol dependent patients benefit from the interventions [16]. A more intensive and prolonged treatment delivered by specialists seems to be needed for the patient group suffering not only from problem drinking, but from alcohol dependence as well [17–20].

In studies on recovery from alcohol use disorders, health problems and hospital admission were among the most cited predictors of recovery [21]. Patients with alcohol use disorders admitted to general hospitals seem much more likely to be motivated for change than a comparable group of alcohol dependent patients in the community [22]. Individuals hospitalized with alcohol use disorders, even just assigned to control groups with no other intervention than assessment, seems significantly more often to remain non-problem drinkers or abstinent after discharge, compared with patients stemming from general practice [23]. In other words; health problems and hospital admission could open a window for changing alcohol consumption. Therefore, general hospitals seem to be in an outstanding position to detect and refer individuals with alcohol use disorders to specialized alcohol treatment.

Several studies have shown that between 36 and 54 % of hospitalized patients do not engage in psychosocial treatment after discharge, and between 20 and 90 % will subsequently relapse back to renewed alcohol abuse [24]. Therefore, barriers to engage in treatment for alcohol use disorders seem to exist. The low number of successful referral rates from inpatient to specialized alcohol treatment represents missed opportunities for patient improvement and cost savings [25].

Lash et al. found that having a therapist complete an aftercare group therapy attendance contract with patients at the completion of inpatient treatment, resulted in significant greater aftercare adherence [26]. In another study, Lash et al. investigated whether a treatment contract drafted by the outpatient therapist at discharge, followed by feedback and reminders in the form of telephone calls and letters, increased the turnout for outpatient group treatment, when compared to a control group who were offered only a treatment contract, drafted by the outpatient therapist. The study showed that significantly more patients in the intervention group began outpatient treatment, and after 12 months significantly more patients in this group were still abstinent/drug-free [27].

The purpose of a referral model is to increase the proportion of patients with alcohol use disorders who start specialized alcohol treatment after being discharged from the hospital. In order to be implemented in the health care system, the model must show an impact on both outcomes for patients and on the overall cost to the health care system. Hence, studies on referral models need to include analyses of cost-effectiveness [28]. In the alcohol treatment field, only few publications describe the cost-effectiveness of treatment alternatives. In a study of an outpatient abuse program in the United States, patients were followed 18 months before and 18 months after starting treatment. After treatment was initiated, a decrease in consumption of health services by 26 % was found [29]. Holder also found a decrease in the use of health services after initiation of treatment [30].

There is a need for developing referral models which can ensure that patients with alcohol use disorders engage in and continue treatment of their addiction after discharge from general hospital. Contracts, prompts and reinforcement developed by Lash et al., are some of the more promising low-cost interventions for increasing participation in outpatient treatment [31]. Attending outpatient treatment is considered essential to successful recovery for patients with alcohol use disorders and it is considered cost-effective for health services. However, only few Danes with alcohol dependence receive outpatient treatment. There is a need to investigate whether a Danish referral model can increase recruitment of alcohol dependent patients from general hospitals to outpatient treatment. This in turn might reduce the economic burden on health care systems and other parts of society and improve the prognosis for the patients.

Inspired by the method of Lash and colleagues, we have developed a Danish referral model called The Relay Model which will address the challenge and establish better referral procedure for dependent drinkers. The purpose of the study is to evaluate The Relay Model in order to assess: i) cost-effectiveness, ii) efficacy and iii) overall societal cost impacts.

## Methods

The study will be conducted as a single-blind pragmatic randomized controlled trial. Patients who are enrolled in the study are included from gastrointestinal, neurological and orthopaedic departments at Odense University Hospital (urban area, serving a population of around 220,000 citizens) and at Aabenraa Hospital (located in rural area serving a population of around 110,000 citizens).

In order to identify the patients a self-report version of the Alcohol Use Disorders Identification Test will be used as a case identification strategy [12]. A completed questionnaire yields a possible score from 0 – to 40 points. According to the cut-off values in the Alcohol Use Disorders Identification Test, a score between 8 and 15 points will be in the hazardous drinking category equal to a medium risk and will benefit from a brief intervention. A score of 15–40 point in the Alcohol Use Disorders Identification Test puts the patient in a harmful drinking or physical dependency category equal to a high risk, where they will benefit from brief intervention and referral to specialist alcohol treatment service.

### Inclusion and randomization

**Fig. 1:**
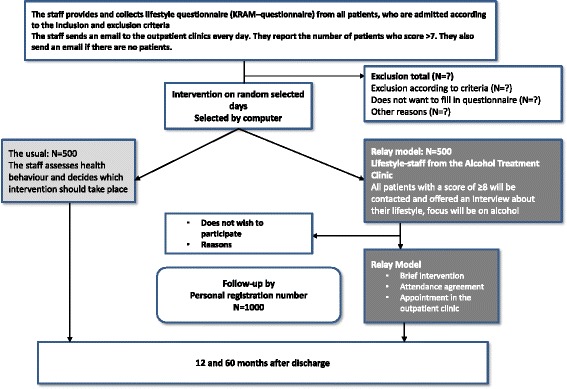
Flow chart

The procedure for inclusion and randomization of the patients is shown in the flow chart (Fig. 1 in Appendix). As a part of standard procedures and quality management, all consecutive patients in the participating departments will be asked to complete a 27-item lifestyle assessment questionnaire concerning smoking, nutrition, exercise and alcohol. The questions on alcohol will include the ten questions in the Alcohol Use Disorders Identification Test. The staff hands out the questionnaire to all patients admitted to the participating departments. Hereafter, the staff collects and scores the questionnaires according to the recommendations [12]. The staff reports the scores to the alcohol treatment clinic, daily. According to randomization, the patient will be allocated to intervention group if the patient meets the following inclusions criteria:

- Not participated in any alcohol-specific treatment for alcohol use disorders in the previous 6 months
- Not psychotic
- More than 18 years of age
- Hospitalized for a minimum of 24 h
- Resident within the uptake area of the involved alcohol treatment clinics
- Willing to participate in the study
- Cognitively and physically capable.

### Allocation concealment

The random allocation sequence of days will be performed by a computer. The randomization will be blinded to the patients as well as the hospital staff. They will not know on which days the alcohol therapist will appear on the ward, clarifying that the sequence will be concealed until interventions are assigned.

### Intervention group

An alcohol therapist will attend the departments on days randomly drawn by a computer. He/she will go through the collected questionnaires, and patients with a score of eight or more in the Alcohol Use Disorders Identification Test will be contacted. The therapist will inform the patients that he/she comes from the outpatient alcohol treatment clinic. The patient will be informed about the study and if he/she consents, the alcohol therapist performs the Relay Intervention including a brief intervention. The therapist will interact with the patient in a non-confrontational and patient-centred manner by using the technique from motivational interviewing [32]. For patients with a score of 16+ in the Alcohol Use Disorders Identification Test, the significance of continuing an outpatient aftercare treatment and suggestion of a contract and an appointment to the alcohol treatment clinic is discussed. The design of the attendance contract is inspired by Lash & Blosser [33], and contains options for attending the outpatient clinic. Finally, patients will be informed that they will most likely meet again; if the patient participates in the outpatient aftercare treatment and that they will receive two letters at two weeks intervals, offering a new appointment should they not meet in the outpatient clinic at the agreed time. The intervention is expected to last 20 min.

### Control group

Patients in this group will receive the usual procedures from the department’s staff.

### Baseline data

To reduce the risk of disturbing the work of the department staff, recruitment data will be limited to data from the Alcohol Use Disorders Identification Test along with few demographic data in the questionnaire.

### Follow-up data

Each Dane has a unique personal registration number [34]. By means of the Danish Civil Registration System, the patients will be followed through the Danish National Patient Registry [35], the Danish Registry of Causes of Death [36] and the Danish National Alcohol treatment Registry [37]. By obtaining data from these registers, we will be able to assess the impact of the interventions on each patient’s use of all possible health care services. Data from all registries will be collected 12 and 60 months after discharge from the departments.

### Objectives

Our primary hypothesis is that the Relay Model will be cost-effective; hence, more patients assigned to the Relay Model will reach the local outpatient clinics. Firstly, we expect that this will lead to a change in alcohol consumption, and further lead to significant reductions in utilization of healthcare services at 12 months follow-up compared to patients assigned with usual referral procedures. Secondly, we expect that significantly more patients assigned to the Relay Model will report for outpatient alcohol treatment when compared with patients assigned to the usual referral procedures. Finally, we expect cost reductions in other societal costs (labour supply, social costs, and traffic accident costs) as well and in public expenditure for income loss compensations at 12 months follow up for patients assigned to The Relay Model.

## Outcomes

### Primary outcome

Health care expenditures 12 months after discharge.

### Data and analysis

Health care expenditure will be measured according to the International Classification of Health Accounts [38], and almost all components can be extracted from the following population registries:

- The Danish National Patient Register [35]
- The Danish National Health Service Register [39]
- The Danish National Prescription Registry [40]
- The Danish Psychiatric Central Research Register [41]

Only ICHA-HC.3 components services of long-term nursing care will not be included in the above mentioned, neither are other national registers, but these will be collected at municipality levels (from Odense Municipal). Whether a particular health care expenditure is related to alcohol will require judgment, which will be undertaken by two clinical assessors who are blinded to patient allocation.

### Secondary outcomes

The percentage of the target group who 30 days after discharge reports at the alcohol treatment clinics and other societal costs including value of alcohol related loss of production.

### Data

IDA National Registry (Integrated Database for Labour market Research), Public expenditures for alcohol related income compensations measured using the DREAM National Registry (Database of Welfare payments and Social benefits) and by PUOB Registry (Database for persons without ordinary occupation).

### Power calculation

In the absence of data from general hospitals, we use data for the power calculation stemming from the study by Parthasarathy et al., who found that outpatient treatment decreased the consumption of health services by 26 % [29]. Primary outcome is the socio-economic costs of alcohol-abuse patients. Based on information from the Danish Institute of Governmental Research we have an estimate of the average total additional expenses for alcohol abuse patients of 88,067 DKK. Treatment for alcohol dependence is expected to lower the additional costs by 26 %. Since the individual excess costs of patients is highly variable with a standard variation of 144,967 DKK, the coefficient of the variation is quite high. Therefore, the power calculation results in a total of about 500 patients in each of the two groups in order to be able to detect the average decrease in total additional expenses of 22,897 DKK, with 80 % probability.

### Blinding

The statistician involved in the data analyses will be blinded to the intervention allocation.

### Analysis

The primary outcome, health care expenditures, will be calculated in two parts. First, a chi-square test will be used to do a crude comparison of the attendance rates of the two groups (intervention and control). Second, health care costs differences will be measured and tested using difference-in-difference analysis. The secondary outcome, societal costs, will also consist of two parts. First, the effect; a marker of reduced alcohol consumption after 12 months (i.e. alcohol related health care contacts), will be assessed in a difference-in-difference analysis. Second, the societal costs will be analysed like health care costs. Cost data are non-Gaussian and non-linear statistical models and bootstrapping techniques will be used in the statistical analyses.

### Ethics

All participating patients with a score of 8+ in the Alcohol Use Disorders Test, suggesting an alcohol use disorder, will be addressed. All data collected in the study will be treated strictly confidentially. No analysis or publication will contain information that allows person-identification. As the study will be based on registers, thus as it does not include human biological material, the study does not need approval from the Regional Scientific Ethical Committees of Southern Denmark [42]. The study was approved by the Danish Data Protection Agency. The study applies to the declaration of Helsinki [43].

### Recruitment

Recruitment started on November 1^st^ 2013. According to the power calculation and based in statistics on admittances, the inclusion will end in the summer of 2016. Follow-up will finish in 2020.

## Discussion

Economic costs are important endpoints in this study. Our economic data and analyses really address several related objectives. Firstly, we believe that economic costs serve as valid indicators of the health and social impacts of our intervention. If the intervention is successful it should show in lower health care expenditures and, in particular, in reduced social costs. In a Danish setting, with an integrated public health sector with basically one third-party payer (the Government) and likewise for delivery of social and welfare services, and with our population registries, we are able to achieve reliable cost data. Secondly, the estimation of economic impacts of our intervention is important in order to politically motivate its implementation and to sort out the financial implications for various public budgets involved.
